# Supplementary material for: SARS-CoV-2 Antigen Detection to Expand Testing Capacity for COVID-19: Results from a Hospital Emergency Department Testing Site
Source: Diagnostics (Basel). 2021 Jul 5;11(7):1211. doi: 10.3390/diagnostics11071211 (PMC8304665; doi:10.3390/diagnostics11071211)
Supplement: Supplementary file 1 [file diagnostics-11-01211-s001.zip › Supplementary_Table_1.pdf]

Table S1 Anticipated NPV and PPV with their 95% CI at indicated COVID-19 prevalence using the STANDARD F COVID-19 Ag FIA assay[a]

Prevalence (%)	COVID-19 area		Non-COVID-19 area	
	PPV% (95% CI)	NPV% (95% CI)	PPV% (95% CI)	NPV% (95% CI)
0.5	17.1 (11.0–25.7)	99.8 (99.8–99.9)	7.21 (4.72–10.9)	99.7 (99.6–99.8)
1	29.4 (19.9–41.0)	99.7 (99.6–99.7)	13.5 (9.1–19.7)	99.4 (99.2–99.5)
2	45.7 (33.5–58.4)	99.3 (99.2–99.5)	**24.0 (16.8–33.1)**	**98.8 (98.5–99.1)**
10	**82.1 (73.3–88.4)**	**96.5 (95.7–97.2)**	63.2 (52.3–72.9)	93.8 (92.1–95.1)
25	93.2 (89.2–95.8)	90.2 (88.1–92.0)	83.7 (76.7–89.0)	83.4 (79.6–86.6)
40	96.5 (94.3–97.9)	82.2 (78.8–85.1)	91.2 (86.8–94.2)	71.5 (66.1–76.4)
80	99.4 (99.0–99.6)	43.4 (38.2–48.8)	98.4 (97.5–99.0)	29.5 (24.5–35.0)

Abbreviations: CI, confidence interval; COVID-19, coronavirus disease 2019; FIA, fluorescent immunoassay; NPV, negative predictive value; PPV, positive predictive value.

[a]Bold indicates values that mirrored those actually observed in the COVID-19 area (pre-test probability, 16.2%) and in the non-COVID-19 area (pre-test probability, 3.7%), respectively.
