# Supplementary figures and images for: SARS-CoV-2 Antigen Detection to Expand Testing Capacity for COVID-19: Results from a Hospital Emergency Department Testing Site
Source: Diagnostics (Basel). 2021 Jul 5;11(7):1211. doi: 10.3390/diagnostics11071211 (PMC8304665; doi:10.3390/diagnostics11071211)

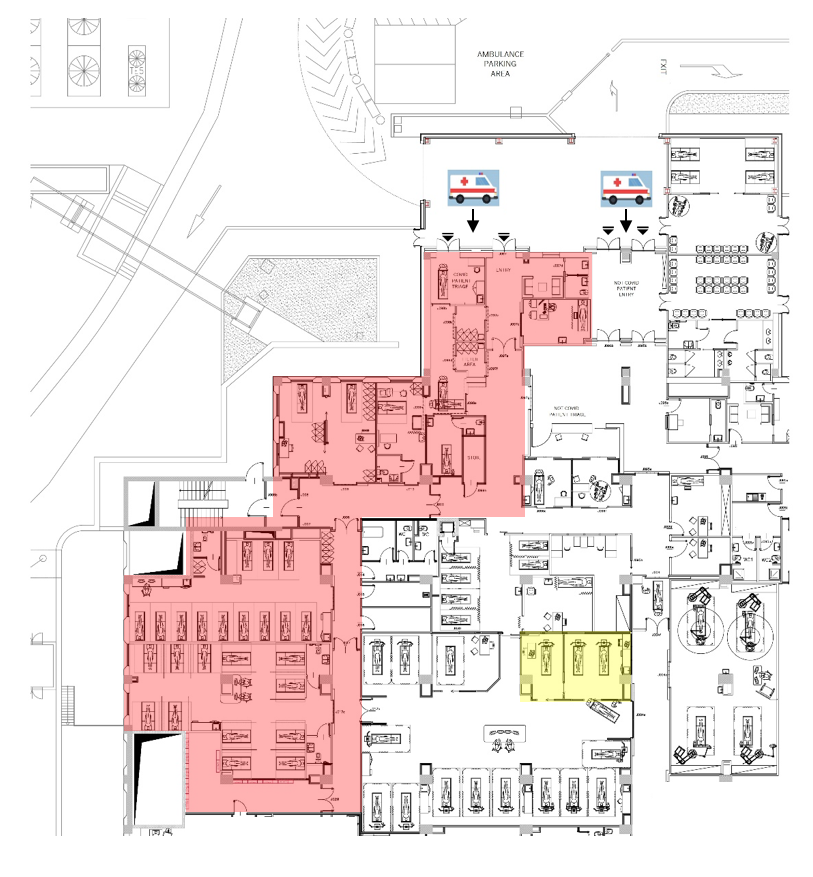

Supplement: Supplementary file 1 [file diagnostics-11-01211-s001.zip › FIGURE_S1.tif]

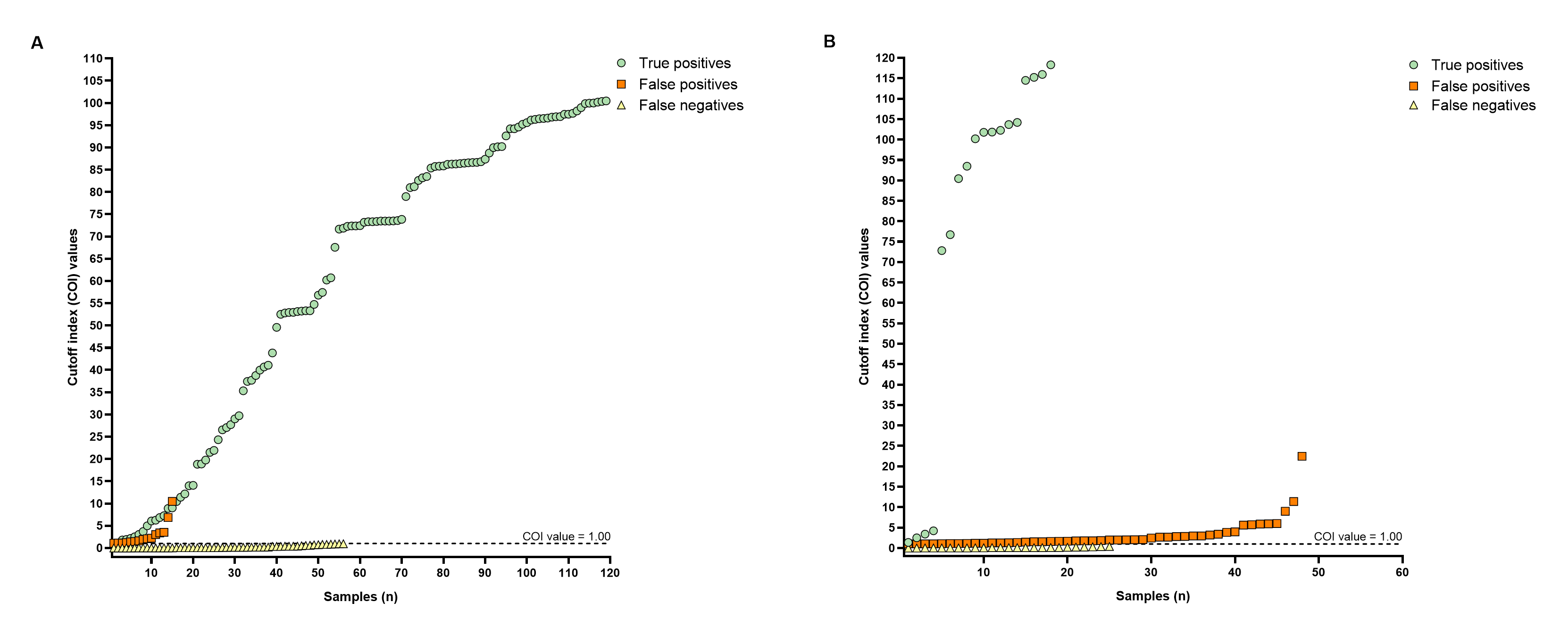

Supplement: Supplementary file 1 [file diagnostics-11-01211-s001.zip › FIGURE_S2.tif]

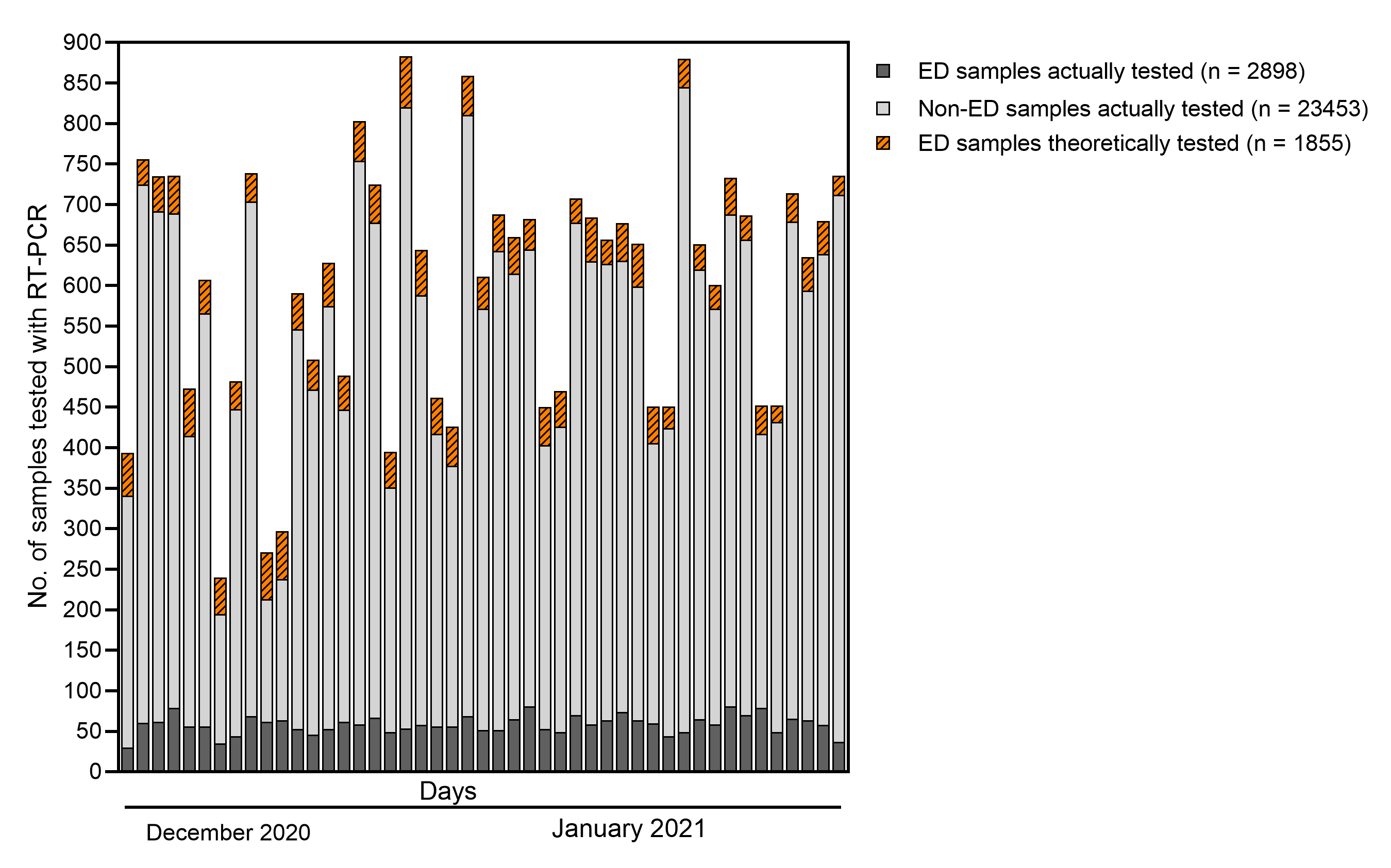

Supplement: Supplementary file 1 [file diagnostics-11-01211-s001.zip › FIGURE_S3.tif]
